# Floating-on-water Fabrication Method for Thin Polydimethylsiloxane Membranes

**DOI:** 10.3390/polym11081264

**Published:** 2019-07-31

**Authors:** Daehan Kim, Sung-Hwan Kim, Joong Yull Park

**Affiliations:** School of Mechanical Engineering, College of Engineering, Chung-Ang University, 84 Heukseok-ro, Dongjak-gu, Seoul 06974, Korea

**Keywords:** polydimethylsiloxane membrane, floating on water, large-area membrane, alternative membrane structure, micron-scale thickness

## Abstract

Polydimethylsiloxane (PDMS) membranes are used in various applications, such as microvalves, micropumps, microlenses, and cell culture substrates, with various thicknesses from microscale to nanoscale. In this study, we propose a simple fabrication method for PDMS membranes on a water surface, referred to as the floating-on-water (FoW) method. FoW can be used to easily fabricate PDMS membranes with thicknesses of a few micrometers (minimum 3 μm) without special equipment. In addition, as the membrane is fabricated on the water surface, it can be easily handled without damage. In addition, alternative membrane structures were demonstrated, such as membrane-on-pins and droplet-shaped membranes. FoW can be widely used in various applications that require PDMS membranes with microscale thicknesses.

## 1. Introduction

Polydimethylsiloxane (PDMS) is a widely used material for microfluidic devices, owing to its simple manufacturing, low cost, transparency, and biocompatible characteristics [1]. PDMS has also been used as a membrane material for a long time, owing to its good permeability, selectivity, and other properties required for membranes [2]. PDMS membranes with various thicknesses, widths, and porosities have been used for various applications, such as microvalves [3,4,5], micropumps [6,7], microlenses [8,9,10,11], and cell culture substrates [12,13]. The spin-coating [14,15], casting [16], and Langmuir–Blodgett methods [17,18] are mainly used for the fabrication of PDMS membranes. Among these, the spin-coating method is the most popular. The spin-coating method involves the spinning of a PDMS solution at a high speed using a spin coater [14]. This method is a very intuitive approach to easily control the membrane thickness according to the rotation speed and rotation time. Through the spin-coating method, undiluted PDMS can produce membranes with thicknesses smaller than 10 μm, while PDMS diluted with a solvent can produce membranes with thicknesses smaller than 100 nm [14,15]. However, the very thin membranes obtained by the spin-coating method cannot be easily handled, as they are attached to the bottom substrate. Therefore, this method is not suitable for easily fabricating a large-area membrane, as a larger force is required for a larger membrane. The casting method involves thinly spreading a PDMS solution, using a casting knife [16]. This can be carried out manually or using a membrane-casting machine. Although the casting method can be used to fabricate a large-area membrane more easily compared to the spin-coating method, it is challenging to maintain a uniform quality of the membrane when the casting is carried out manually. If a membrane casting machine is used, the membrane quality is uniform, but additional equipment is required. Furthermore, the Langmuir–Blodgett method can be used to fabricate a membrane by collecting the separated molecules of materials on water using a moving barrier [17,18]. Owing to the self-assembly effect, a well-aligned membrane can be fabricated on a molecular basis. This method is not widely used to fabricate PDMS membranes, but rather biomimetic membranes using organic materials [19], as it requires expensive equipment to control the molecular level thickness of the membrane. In summary, the above-mentioned three methods require additional equipment and skilled researchers, and still cannot be used to easily fabricate large-area membranes.

In this paper, we propose a method to fabricate a thin (<10 μm) PDMS membrane by placing a liquid PDMS solution on the surface of water and curing the PDMS solution. This membrane fabrication method, utilizing the characteristics of the two immiscible phasic fluids (water and PDMS), is referred to as the floating-on-water (FoW) method. The fabrication process is simply done by only using a hot plate. Briefly, after filling a Petri dish with water, the PDMS solution is dropped and heated to obtain various membranes with thicknesses of a few to tens of micrometers. In addition, FoW-based membranes can be more easily handled than those obtained by spin-coating [15] on solid substrates because there is no risk of tearing or breakage as the membrane floats on water when the FoW process steps are completed. The immiscible characteristics of water and PDMS have been utilized to fabricate microstructures [20]; however, to the best of our knowledge, this is the first report on fabricating membranes. The FoW method, which can be used to fabricate thin PDMS membranes without the need of expensive equipment, is expected to be a useful technique for researchers in various applied research fields (e.g., microvalves [3,4,5], micropumps [6,7], etc.) where membranes with various thicknesses are used.

## 2. Materials and Methods

### 2.1. Fabrication

**Figure 1:**
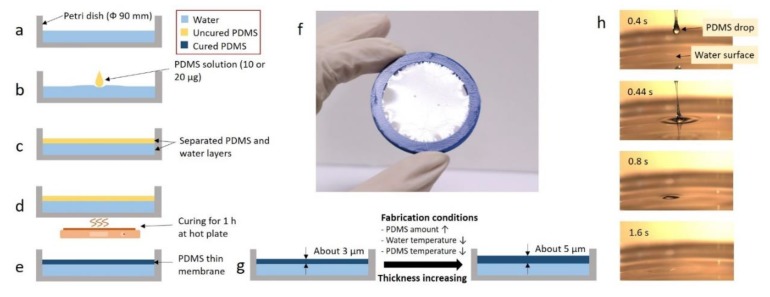
Polydimethylsiloxane (PDMS) membrane fabrication using the floating-on-water (FoW) method. (**a**) 50 mL of water was added to a 90-mm Petri dish. (**b**) 10 or 20 μg of the PDMS solution was dropped onto the water surface. (**c**) PDMS solution spread over the water surface. (**d**) Petri dish was heated on a hot plate at 40 or 80 °C. (**e**) PDMS solution was cured, yielding the PDMS membrane. (**f**) Image of the fabricated PDMS membrane lifted by a support ring. (**g**) Membrane thickness variations under three different conditions. (**h**) High-speed camera images of the PDMS drop spreading on the water surface. In approximately 2 s, the PDMS droplet loses its shape and spreads on the water surface.

The PDMS base and PDMS curing agent (Sylgard 184, Dow Corning, Midland, MI, USA) were thoroughly mixed at a ratio of 10:1 (*w*:*w*). The PDMS solution was then vacuum-treated in a desiccator for 30 min to remove bubbles entering the PDMS solution, and 50 mL of water was used as a substrate in a Petri dish with a diameter of 90 mm (Figure 1a). Then, 10 or 20 μg of the PDMS solution was dropped onto the water surface (Figure 1b). As PDMS is lighter than water and immiscible with water, the dropped PDMS solution floated and spread on the water surface [21]. The PDMS solution gradually spread over the water surface quickly (<30 s) and eventually covered the water surface (Figure 1c,h). When the Petri dish was placed on a hot plate at 40–80 °C for 1 h (Figure 1d), the well-spread PDMS solution was cured, yielding a thin PDMS membrane with a thickness smaller than 10 μm (Figure 1e,f). The moment of the PDMS dropping was filmed with a high-speed camera (FASTCAM Mini UX100, Photron, Tokyo, Japan) (Figure 1h, Videos S1 and S2). When the PDMS contacted the water surface, it bounced a little due to the immiscibility and surface tension. Then, the PDMS droplet lost its visibility as it spread over the water surface. As a result of measuring the movement of the PDMS through the images, it was found that the PDMS spread over the water surface by 16.5 mm in 1 s. Therefore, a minimum of approximately 5.5 s is required to cover the diameter of a 90-mm Petri dish. The above experiment was carried out when the temperature of the PDMS and water were at room temperature (20 °C).

### 2.2. Membrane Thickness Control

The thickness of the membrane fabricated by FoW can be controlled by various factors during the fabrication (Figure 1g). The membrane thickness varies with the amount of PDMS solution. Therefore, the amount of PDMS solution is the first factor that can be used for membrane thickness control. As the water surface area is already determined by the size of the container (Petri dish diameter: 90 mm), the thickness of the membrane is determined by the volume of PDMS dropped on the water surface. A 1000-μL pipette tip (T-1000-B, Axygen Scientific, New York, NY, USA) was used to control the amount of PDMS solution. After loading PDMS in the pipette tip, the tip was vertically placed above the water surface to naturally drop the PDMS solution onto the water by gravity. Using this method, the thickness of the PDMS membrane was obtained by dividing the amount of PDMS solution into 10 μg (one drop) and 20 μg (two drops). Another control factor determining the thickness of the PDMS membrane is the water temperature. The temperature has a significant influence on the curing time of PDMS. To investigate the change in the membrane thickness according to the water temperature, PDMS was cured for 1 h on a hot plate at different temperatures of 40 and 80 °C. The final parameter is the initial temperature of the PDMS solution, as the temperature of the liquid-phase PDMS affects the initial viscosity. When the temperature of the PDMS increases, the viscosity decreases, and vice versa [22]. To investigate the change in membrane thickness according to the temperature of the PDMS solution, the analysis was carried out at temperatures of 20 and −15 °C (the sample was stored in a freezer for one day). When the membrane was fabricated, the temperature of the PDMS solution could not be maintained because the PDMS solution was heated by the hot plate, so, the PDMS solution temperature condition can be regarded as the "initial" temperature condition. Therefore, including the control condition, four control factors were evaluated, as presented in Table 1. Five membranes for each condition, i.e., 20 membranes in total, were fabricated and analyzed using scanning electron microscopy (SEM).

As the membrane is very thin, the amount of PDMS solution supplied onto the water must be finely adjusted. The required amounts of most solutions can be supplied by a syringe; however, as PDMS has a high viscosity, it is challenging to provide the required amount using a syringe. Therefore, in FoW, a constant amount of PDMS solution was supplied using a pipette tip rather than a syringe. When the pipette tip was put in the PDMS solution and lifted, the PDMS solution gathered at the pipette tip and dropped by gravity. If the amount of PDMS deposited on the pipette tip is too small, the PDMS solution does not fall, owing to the viscosity, while if the amount of PDMS exceeds the threshold, the PDMS solution begins to fall, owing to its weight. The amount of PDMS at the threshold is always the same so that a constant amount of PDMS solution can be supplied over the water surface. PDMS dropping experiments were conducted using 10, 100, and 1000-μL pipette tips to see if the pipette tips could continuously supply the proper amount of PDMS. After the PDMS solution was sufficiently filled on the surface of the pipette tip and naturally fell, the weight of one drop of PDMS was measured using a precision balance. The experiment was repeated four times for each pipette tip size.

### 2.3. Membrane Strain Rate Test

A membrane strain rate test was performed to measure the mechanical properties of the FoW membrane. The 5 and 11-μm-thick membranes were attached to a ring-shaped holder of 40-mm-diameter and pulled until they were torn. The ring-shaped holder was three-dimensionally (3D) printed with acrylonitrile butadiene styrene material (Supplementary Figure S1). In another approach, experiments were conducted to deform the membrane using the weight of water. Water was added dropwise to the 3, 5, or 11-μm-thick membranes and the length of the membranes were measured when they were torn. The volume of one drop of water was 100 μL. An image was taken from the frontal view and the two-dimensional length of the membrane was measured using an image analysis software, ImageJ.

### 2.4. Statistical Analysis

A one-way analysis of the variance was performed on the measured membrane thickness data. A *p*-value smaller than 0.05 was considered statistically significant.

### 2.5. ATR-FTIR Surface Chemical Analysis

The attenuated total reflection Fourier transform infrared (ATR-FTIR) test was performed to determine if unintended chemical reactions occurred during the FoW process, using a Nicolet 6700 FTIR spectrometer (Thermo Scientific Inc., Madison, WI , USA). The test was performed on the FoW membrane (5-μm-thick) and spin-coated PDMS membrane (100-μm-thick). The FTIR spectra were recorded at 2 cm^−1^ intervals between 4000 and 400 cm^−1^ and each spectrum was scanned 32 times. The results were analyzed using OMNIC software (v. 8.2, Thermo Scientific Inc.).

### 2.6. Topography Analysis by AFM

An atomic force microscopy (AFM) test was performed to investigate the nanoscale surface topography of the FoW membrane. The 2D and 3D surface topography and surface roughness were obtained using nanoman II (Veeco, Santa Barbara, CA , USA) in tapping mode. Experiments were performed with an antimony-doped silicon tip (radius of 8 nm, height of 10 μm, RTESP-300, Bruker, Billerica, MA, USA) with a scan area of 1 × 1 μm^2^ and a scan rate of 1 Hz. The mean roughness (*R_a_*), root mean square roughness (*R_q_*), and maximum roughness depth (*R_max_*) were obtained by the AFM test.

## 3. Results and Discussion

### 3.1. Membrane Fabrication

The FoW method was successfully used to fabricate various membranes. A small amount of PDMS solution was dropped onto water to fabricate a thin membrane floating on the water. The thickness of the membrane fabricated by FoW was adjustable by varying the conditions. We used three parameters to control the membrane thickness: (1) amount of PDMS solution, (2) temperature of water, and (3) temperature of PDMS solution (see Table 1). The PDMS solution immediately spread over the surface and formed a membrane within 1 h of being dropped onto the surface of the water at 80 °C. However, the transparent membrane formed on the surface of the water could not be easily distinguished by the naked eye. The formation of the membrane could be confirmed by a water drop or spray (Supplementary Figure S2). The resulting membrane is significantly easier to handle than those obtained by other membrane fabrication methods as it floats on the water. As the adhesion between the membrane and water is not strong, the membrane can be easily moved to a desired site. The membrane-handling is presented in detail below. It is worth noting that if the bottom is tilted or vibrates, the membrane may not be properly formed.

**Figure 2:**
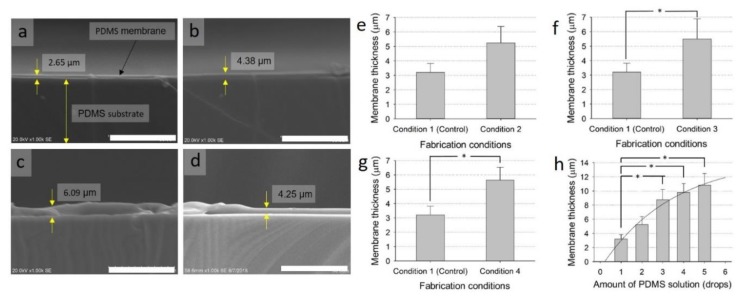
SEM images of the PDMS membrane fabricated by FoW and membrane thickness graphs. (**a**–**d**) Fabrication conditions 1–4; the membrane thicknesses are 2.65, 4.38, 6.09, and 4.25 μm, while the mean thicknesses are 3.213, 5.241, 5.490, and 5.640 μm, respectively. The scale bars in (**a**–**d**) correspond to 50 μm. The uneven portion of the membrane was made while preparing samples for SEM imaging and was excluded from the measurement. (**e**–**g**) Membrane thickness graphs compared to condition 1 (control) for the different experimental conditions; * *p* < 0.05 (*n* = 5). (**h**) Membrane thickness variation with respect to the amount of PDMS solution (one (=10 μg) to five (=50 μg) drops); * *p* < 0.05 (*n* = 5). The solid-line curve is achieved by the regression analysis.

For the control group (condition 1, Table 1), the control factors were: one drop of PDMS solution (10 μg), water temperature of 80 °C, and room-temperature (20 °C) PDMS solution. A very thin membrane (several micrometers) was obtained using the control conditions of FoW (Figure 2a). The mean membrane thickness for condition 1 was 3.213 ± 0.608 μm (*n* = 5, Figure 2e). Furthermore, the amount of PDMS was changed (condition 2, Table 1) for comparison with the control sample (condition 1). The amount of PDMS was doubled (two drops, 20 μg); the other parameters (water and PDMS solution temperatures) were fixed. The membrane thickness changed according to the amount of PDMS placed on top of the water. The mean membrane thickness for condition 2 was 5.241 ± 1.135 μm. Compared with that for condition 1, the membrane thickness increased by approximately 63%, less than twice the thickness. This can be explained, as a considerable amount of PDMS solution floating on the water near the Petri dish wall tends to stick to the wall, owing to the surface tension (Supplementary Figure S3). This phenomenon is visible with a microscope or even with the naked eye. This unwanted capillary effect has a strong influence on the thickness of membranes near walls, which might be solved by using PDMS-repellent materials, if available. The membrane thickness near walls, therefore, is different from that of the central part of the membrane, as the capillary effect cannot be prevented at present; thus, all the data were tested for the central part. The third control parameter was the water temperature (condition 3, Table 1). The temperature of the water (50 mL) was controlled using a hot plate. The hot plate heated the water to the target temperatures of 80 °C for the control condition (condition 1, Table 1) and 40 °C for the test condition (condition 3). The temperature of the water had a considerable effect on the curing time of the PDMS solution, as the PDMS solution drop is small and the fabricated PDMS membrane is thin. The measured mean membrane thickness for condition 3 was 5.490 ± 1.392 μm (Figure 2c,f). This shows a large increase in the thickness compared with that for the control condition. Particularly, the standard deviation significantly increased, which could be attributed to the delay in the curing time of the membrane, as the curing temperature was decreased under condition 3. The final membrane thickness control parameter is the initial temperature of the PDMS solution (condition 4, Table 1). PDMS solution batches prepared for this experiment were placed in a freezer at −15 °C for one day and at room temperature (20 °C). The mean membrane thickness for condition 4 was 5.640 ± 0.889 μm (Figure 2d,g). The membrane thickness tended to increase, as under the other conditions. The origin of the increase in the thickness for condition 4 could be attributed to the change in viscosity of PDMS, depending on its temperature. The temperature of the PDMS solution affects its viscosity [22]. When the PDMS solution droplet falls onto the surface of the water, it spreads on the surface; the rate of spreading depends on the viscosity. The temperature of the PDMS solution spreading on the water is affected by the water temperature; however, the initial viscosity of PDMS is still an important control factor determining the thickness of the PDMS membrane.

Among the control schemes of the membrane thickness, the most direct and simple control parameter is the number of PDMS solution drops. To clarify its effect on the membrane thickness, an additional set of experiments were performed by applying three, four, and five PDMS solution drops (five samples for each). The thickness of the membrane was measured according to the amount of PDMS (one (=10 μg) to five (=50 μg) drops). This method could be used to change the membrane thickness in a wide range, compared with the variations in the water and PDMS temperatures. When five drops (50 μg) of PDMS solution were dropped, a membrane with a thickness of approximately 11 μm was obtained (Supplementary Figure S4), which is more than three times thicker than the control membrane (condition 1) (Figure 2h). The regression model of the membrane thickness according to the amount of PDMS solution droplet is as follows:

(1) $$y=a+b\left(1-e^{cx}\right)$$

where *a* is −0.9359, *b* is 15.5315, *c* is −0.2921, *x* is the number of PDMS drops (1 drop = 10 μg), and *y* is the membrane thickness (μm). The regression model has three parameters, exponential rise to maximum using statistical analysis software SigmaPlot 12 (Systat Software Inc., San Jose, CA, USA).

To evaluate whether a constant amount of PDMS solution can be precisely supplied, the amount of supplied PDMS was measured on a precision balance using pipette tips with various sizes (Supplementary Figure S5). With 10-, 100-, and 1000-μL pipette tips, the weight of one drop of PDMS solution was measured four times, yielding averages of 6, 8, and 10 μg and standard deviations of 0.122, 0.515, and 0.485 μg, respectively (Supplementary Figure S5). Based on these results, which exhibited slight differences between the experiments, we concluded that a constant amount of PDMS solution could be supplied using a pipette tip. The 1000-μL pipette tip was used in the further experiment to provide a larger amount of PDMS at once. The area of the membrane fabricated by FoW is equal to that of the surface of the water (=top view area of the container holding the water) covered by the PDMS solution. However, unless the amount of PDMS is sufficient to cover the surface of the water, a membrane may not be formed on an arbitrary part of the water surface (Supplementary Figure S6). An amount of 10 μg of PDMS solution supplied by the 1000-μL pipette tip is not sufficient to cover a 150-mm Petri dish; however, it is sufficient to cover a 90-mm Petri dish.

### 3.2. Membrane Strain Rate

**Figure 3:**
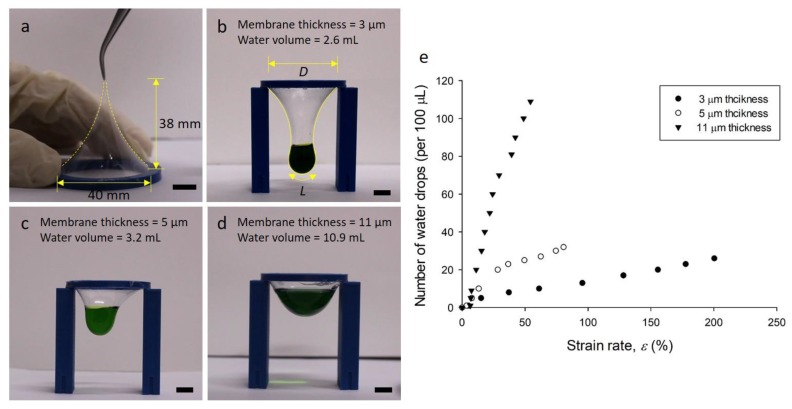
Stretching properties of the membrane. (**a**) Membrane with a diameter of 40 mm and thickness of 5 μm, stretches very flexibly up to 38 mm. (**b**) 3-μm-thick membrane, fabricated by one drop of PDMS solution, can contain 2.6 mL of water. (**c**) 5-μm-thick membrane, fabricated by two drops of PDMS solution, can contain 3.2 mL of water. (**d**) 11-μm-thick membrane, fabricated by five drops of PDMS solution, can contain 10.9 mL of water. The scale bars in (**a**–**d**) correspond to 10 mm. (**e**) Strain rates of the membranes as a function of the number of water droplets. The volume of one water drop is 100 μL.

The properties of PDMS membranes vary with their thicknesses [23,24]. In this regard, we carried out a membrane strain test using membranes with various thicknesses fabricated by FoW. When the 5-μm-thick membrane (Condition 2) was pulled upward using the tweezers, the membrane was extended by 38 mm before breakage (Figure 3a). We carried out the same experiment on the 11-μm-thick membrane, which was extended by 19 mm, half of the extended length of the 5-μm-thick membrane (Supplementary Figure S7). This experiment demonstrated that a thinner membrane can be extended to a higher degree. For further investigation, water was dropped on the membrane specimen to observe the deformation and calculate the strain rate. Membrane specimens with three different thicknesses of 3, 5, and 11 μm were prepared. The 3 and 5-μm-thick membranes could be obtained by conditions 1 and 2 (Table 1), respectively, while the 11-μm-thick membrane could be obtained by five drops of PDMS solution. When water was added dropwise, the membrane was continuously deformed, proportional to the applied mass of water. The water droplets were dropped on the membrane specimens with thicknesses of 3, 5, and 11 μm until the membranes were torn; the strain was calculated by [14]:

(2) $$\varepsilon =\frac{L-D}{D}$$

where *ε* is the strain of the membrane, *L* is the length of the membrane stretched by water (the length *L* of the membrane represents the two-dimensional length in the frontal view), and *D* is the original diameter of the membrane. The 3-μm-thick membrane contained 2.6 mL of water; its strain rate was 200%. The 5-μm-thick membrane contained 3.2 mL of water and had a strain rate of 80%. Meanwhile, the membrane with a thickness of 11 μm contained 10.9 mL of water and its strain rate was 54%. These strain rates are significantly different. The experimental results show that a thicker membrane can contain a larger amount of water, but the strain rate decreases. The very high strain rate of the thin PDMS membrane is useful for various applications. In addition, the feature that the strain rate varies with the thickness can be utilized for applications. The ratio of PDMS base and curing agent has a great influence on the elastic modulus of PDMS [25]. It also affects the permeability of the PDMS membrane [26]. The reason for increasing the permeability is that the larger the ratio of the PDMS base and the curing agent, the greater the elastic modulus, so the micropores inside the membrane are easily changed. In this study, the ratio of PDMS base to curing agent was fixed at 10:1 (w:w), but the ratio could be modified to change the properties of the membrane.

### 3.3. Alternative Membrane Structures

**Figure 4:**
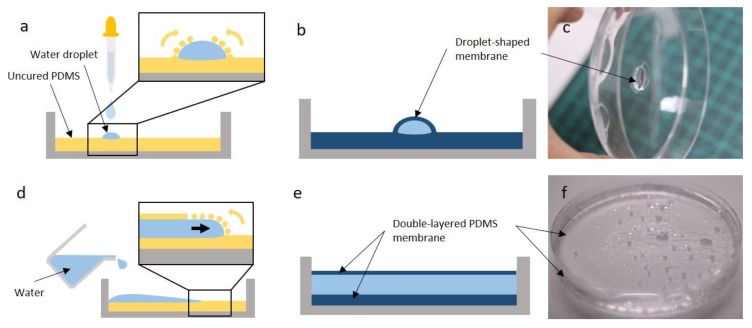
Schematics and images of droplet-shaped and double-layered PDMS membranes. (**a**) When the water droplet was dropped on the coated PDMS solution, the PDMS solution slowly covered the whole water droplet surface. (**b**) Upon the curing of the PDMS solution, a droplet-shaped PDMS membrane formed. (**c**) Image of the droplet-shaped PDMS membrane. The water droplet does not fall down, even if the Petri dish stands vertically. (**d**) Upon the pouring of water on the coated PDMS solution, a small amount of PDMS solution creeps up the water surface as the specific gravity of PDMS is slightly lower than that of water. (**e**) The double-layered PDMS solution on the top and bottom of the water is cured, yielding a double-layered PDMS membrane. (**f**) Image of the double-layered PDMS membrane. Water droplets sprayed onto the membrane indicate the presence of the membrane.

One of the advantages of FoW is that not only flat shapes, but also curved shapes, can be fabricated. A unique characteristic of FoW is the buoyancy of the PDMS solution, implying that the fabricated membrane has the geometry of the water surface, which can be flat or curved. As an example, if a small amount of water that does not cover the whole surface of the Petri dish is poured on the pre-layered PDMS solution, the water forms a water droplet because of the surface tension (Figure 4a). Subsequently, the pre-layered PDMS solution around the droplet climbs over the water droplet surface. The droplet-shaped membrane can be obtained by curing the PDMS solution over the water droplet surface (Figure 4b). Moreover, the PDMS solution climbs over the water droplets, owing to the low specific gravity of PDMS. Using this mechanism, droplet-shaped membranes of various sizes and shapes can be easily fabricated.

Another membrane structure is the double/multiple-layered membrane. By carefully introducing water on the uncured PDMS solution coated at the bottom of the Petri dish, followed by curing, a double-layered PDMS membrane was successfully fabricated (Figure 4d). This was achieved by the difference in specific gravity between the PDMS solution (lighter) and water (heavier). A small portion of the uncured PDMS solution on the bottom slowly moves to the surface of the water and forms another thin PDMS film. The PDMS coated on the bottom and that on the water surface form a membrane, yielding a double-layered membrane. If a small droplet of water is deposited instead of a large amount of water on the uncured spin-coated membrane, the PDMS rises on the droplet to form a droplet-shaped membrane.

**Figure 5:**
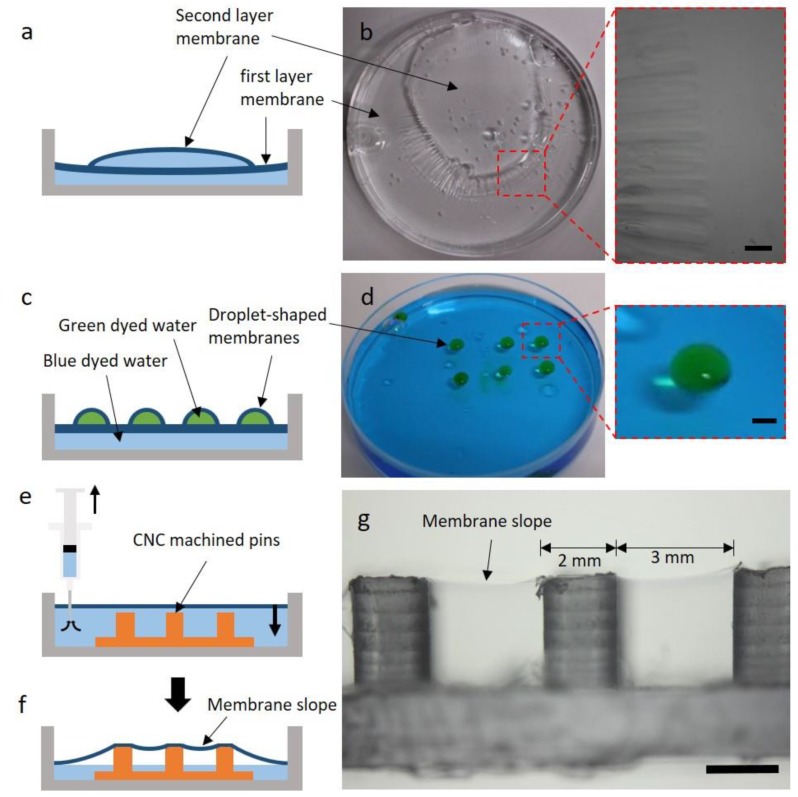
Schematics and images of double-layered and droplet-shaped PDMS membranes and the membrane-on-pins structure. (**a**) Double-layered membrane fabricated upon the addition of water and PDMS solution on the PDMS membrane fabricated by FoW. (**b**) Image of the double-layered PDMS membrane. The area of the second layer of the membrane is not equal to that of the whole surface of the Petri dish, as the first layer membrane is sagged by the weight of the water. A wrinkled morphology is observed around the face of the two membranes, owing to the weight of the water inside the top membrane. The scale bar corresponds to 1 mm. (**c**) When the water droplet is dropped onto a partially cured FoW membrane, droplet-shaped membranes are formed. (**d**) The water below was dyed blue, while the water droplet was dyed green. The scale bar corresponds to 2 mm. (**e**) A PDMS membrane was prepared in the same manner as above. Pins were then placed under the PDMS membrane and water was removed by the syringe. (**f**) The membrane is descended, yielding a membrane-on-pins structure. (**g**) Microscopy image of membrane-on-pins. The scale bar corresponds to 2 mm. The furrow-like membrane slope is observed between the pins.

Further alternatives can be obtained by combining the above methods. Another type of double-layered membrane can be fabricated by pouring a desired amount of water onto the membrane fabricated by FoW, and dropping the PDMS solution on the newly added water (Figure 5a). However, in this case, the bottom membrane was sagged by the weight of the added water and created wrinkles around the bottom membrane (Figure 5b). Droplet-shaped water capsules can also be fabricated using the same approach, by placing water droplets on the fabricated membrane or by dropping water droplets on the PDMS solution spread on the water but not fully cured, which enables the uncured PDMS solution to creep up the water droplets (Figure 5c). Another variation can be created using a pin array fabricated by a computer numerical control (CNC) machine (Figure 5e). As the pin array (3 × 3, in this study) was placed under the membrane generated on the water surface, which was then gradually descended by water removal (Figure 5f), a unique membrane-on-pins structure formed (Figure 5g). Between the pins, a furrow-like membrane structure was created (Figure 5g). There are several factors related to the thickness of the droplet-shaped membrane and the double-layered membrane. In the droplet-shaped membrane, the size of the droplet and the curing temperature are expected to have the greatest effect on the thickness. In a double-layered membrane, the strength of pouring water on the uncured PDMS layer and the time it takes to cover the surface of the water will be important for determining the membrane thickness. However, it is difficult to fine-tune and experiment with these conditions, and it is also difficult to accurately measure the thicknesses of the fabricated droplet-shaped membrane and double-layered membrane. Therefore, further research is required for the applications of these membrane types.

### 3.4. Large-Area Membrane

**Figure 6:**
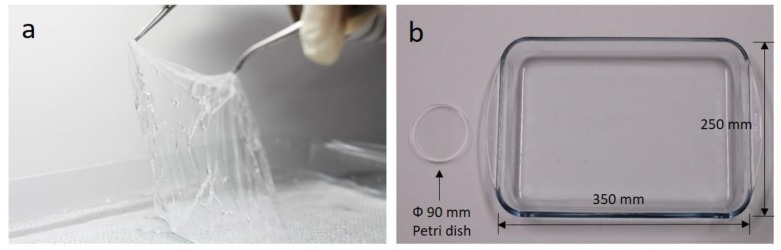
Large-area membrane fabrication. (**a**) The larger membranes can be easily fabricated if a sufficient amount of PDMS solution is applied. (**b**) A large glass container (350 × 250 mm^2^) was used to fabricate large FoW membranes.

Unlike other membrane fabrication methods mentioned in the introduction section, FoW can be used to easily fabricate a large-area membrane. To fabricate a large-area membrane using the spin-coating method, large substrate and power to rotate the large substrate are required. However, FoW requires only larger water containers. We fabricated a membrane with a large area of 350 × 250 mm^2^ (Figure 6), 13.7 times larger than that with the diameter of the Petri dish (90 mm). According to the principles of FoW, there is no limit to the area of the membrane as long as the container is sufficiently large. It should be noted that the amount of PDMS solution needed to obtain a specific thickness may differ from that for the membrane fabricated in the Petri dish, as a larger amount of PDMS solution can attach to the container wall surface.

### 3.5. Membrane Handling

As mentioned above, as the membrane fabricated by FoW is transparent, it is challenging to visually verify the formation of the membrane on the water surface. However, there is a risk of tearing if the membrane formation is verified physically using tools such as tweezers. The method used in this study to identify small droplets involved dropping or spraying water on the fabricated membrane (Supplementary Figure S2). If the water drops are absorbed in the underlying water and disappear, the membrane is not fabricated; while if the water drops preserve their shapes, the membrane is fabricated. This simple technique enables quick and easy verification of the membrane formation without damaging the membrane.

The fabricated PDMS membrane is very thin; thus, is prone to damage during handling. Several other papers have introduced handling methods for very thin membranes. For example, a donut-shaped PDMS ring with a thickness of 1–2 cm was prepared to handle a spin-coated PDMS membrane on a wafer [14]. The PDMS ring was attached to the membrane by a plasma treatment, then used to peel the membrane from the solid substrate. However, in this method, the membrane is excessively stretched or torn during the peeling-off process. It is often the case that membrane fabrication is a part of a system fabrication, for which no specific handling device is necessary [15]. Therefore, it varies between different cases; thus, a standardized/universal membrane fabrication method cannot be developed. Unlike these membrane fabrication methods, FoW enables simple and force-free handling as the fabricated membranes floating on the water surface barely contact other solid objects. In FoW, the water serves as a substrate, and the water and PDMS do not adhere to each other, so the membrane can be easily and simply handled, preventing undesired deformation and damages. For further convenience in membrane handling, a scooper device (Supplementary Figure S1) made of PDMS or plastic can be used. The scooper can be moved over the floating membrane, as well as under the membrane, as the adhesion between the scooper and membrane is stronger than that between the water and membrane. Therefore, the membrane can easily stick to the scooper and be controlled.

### 3.6. Surface Analysis of FoW Membrane

**Figure 7:**
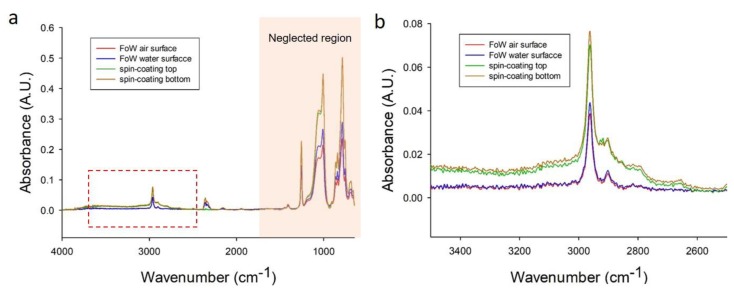
ATR-FTIR graph of FoW membrane and spin-coated membrane. As the FoW membrane is fabricated on the water surface, the upper side of the membrane touches the air and the lower side of the membrane touches the water. Therefore, the top (red) and bottom (blue) surfaces of the FoW membrane were analyzed, respectively. The top (green) and bottom (brown) surfaces of the spin-coated membrane were also analyzed as comparison conditions. (**a**) Graph in full range of wavenumber. (**b**) Graph in the focused region near the wavenumber of 3000 cm^−1^.

Unlike conventional membrane fabrication methods, the FoW method utilizes water. It is necessary to confirm whether any chemical changes occurred on the membrane surface. The surface of the membrane (5 μm thick), made via FoW using ATR-FTIR, was analyzed and compared with the spin-coated membrane (100 μm thick) (Figure 7a). As the FoW membrane is fabricated on the water surface, the upper side of the membrane touches the air, while the lower side is in contact with the water. Therefore, the top (red) and bottom (blue) surfaces of the FoW membrane were analyzed, respectively. The top (green) and bottom (brown) surfaces of the spin-coated membrane were also analyzed as comparison conditions. As peaks in the wavenumber range of lower than 1500 cm^−1^ are not usually considered in polymer surface analysis, the peaks near the wavenumber of 3000 cm^−1^ were carefully examined (Figure 7b). The peak positions of the four conditions were the same at 2962 cm^−1^. The tendency of the graphs among the top and bottom surfaces of the FoW membranes was the same, which is also true for those among the top and bottom surfaces of spin-coated membranes. However, the absorbance magnitudes between the FoW membrane and the spin-coated membrane were different. The difference in the absorbance magnitude may have occurred due to membrane thickness. However, because the difference in size is not a difference in chemical composition itself, the FoW membrane can be used in the same way as those made by other conventional methods.

**Figure 8:**
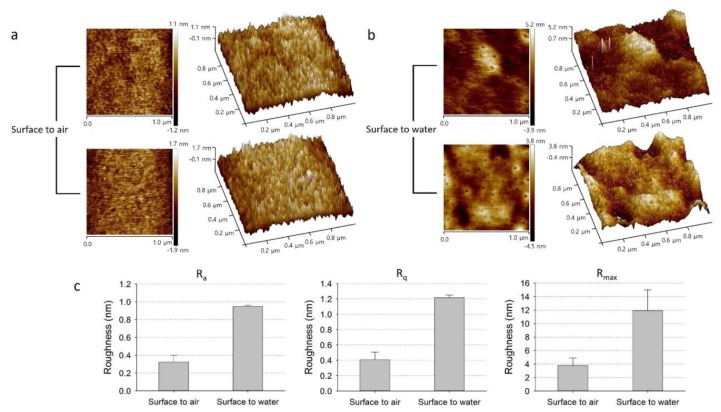
2D and 3D AFM images of FoW membrane surfaces: (**a**) Surface-to-air and (**b**) surface-to-water. The difference in roughness between the surface to air and the surface to water of the membrane is significantly large. (**c**) The graphs of *R_a_*, *R_q_*, and *R_max_* show different levels of roughness between the two surfaces; on average, the roughness of the surface-to-water is approximately three times higher than that of the surface-to-air. The average values of *R_a_*, *R_q_*, and *R_max_* of surface to air are 0.322, 0.409, and 3.77 nm and standard errors of 0.077, 0.098, and 1.11 nm, respectively. The average values of *R_a_*, *R_q_*, and *R_max_* of the surface-to-water were 0.947, 1.215, and 11.925 nm and standard errors of 0.015, 0.035, and 3.075 nm, respectively (*n* = 2).

The surface topography of the FoW membrane was obtained using AFM. The AFM experiments were conducted on both the surfaces of the FoW membrane, in contact with the air and water, respectively. On the surface-to-air, the average *R_a_*, *R_q_*, and *R_max_* were 0.322, 0.409, and 3.77 nm, respectively (Figure 8a). On the surface-to-water, the average *R_a_*, *R_q_*, and *R_max_* are 0.947, 1.215, and 11.925 nm, respectively (Figure 8b). The roughness of the FoW membrane is not significantly different from the roughness of the membrane obtained by other methods; a 50-μm-thick PDMS membrane was fabricated using the spin-coating method, and obtained *R_a_* of 1.593 nm [27], and a collagen-coated PDMS membrane was fabricated using the casting method, which obtained *R_a_* of 0.58 nm [28]. The surface roughness on the surface-to-water was significantly higher than that of the surface-to-air. All values of the water surface were larger than the values of the surface to air. On average, *R_a_*, *R_q_*, and *R_max_* increased by 216, 194, and 197%, respectively, on the surface-to-water (Figure 8c). There could be several reasons why the roughness here is larger than that on the surface to air. First, there was a difference in the roughness due to the thermal fluctuations of air and water. Water is denser in molecular structure than air. Therefore, PDMS and water are more closely tied than PDMS and air, and PDMS is more susceptible to thermal fluctuation by water. This difference in thermal fluctuation would result in a difference in roughness between the surface-to-water and surface-to-air sides. The difference in thermal fluctuation would have been greater because water is heated during membrane fabrication. Second, the roughness would have changed due to a larger scale of water movement than the thermal fluctuation. As the water heats, the temperature difference between the top and bottom of the water causes convection inside the water, which causes a wave of the surface of the water. Third, the humidity is controlled to prevent evaporation of water, but it cannot completely prevent evaporation, so slight evaporation can affect the membrane surface. For these reasons, the roughness on the surface-to-water would have been larger than the roughness on the surface-to-air. The surface roughness can affect the adhesion between the membrane and other devices. As a result, the surface-to-air is less rough than the surface-to-water; therefore, it is better to use the surface-to-air of the membrane for bonding with other surfaces. However, both surfaces can be utilized for general purposes, as the maximum difference in roughness (*R_max_*) is in the order of several nanoscales (Figure 8c).

## 4. Conclusions

In this paper, we proposed a novel FoW method to fabricate a PDMS membrane on a water surface and characterized the membrane thicknesses under various fabrication conditions. The membrane thickness (approximately 3–12 μm) depended on the PDMS amount, water temperature, and PDMS temperature. The FoW, which is a significantly simpler method than conventional membrane fabrication methods, was successfully used to fabricate a membrane with a thickness similar to those of fabricated by the conventional methods. The FoW method does not require expensive equipment, and even a non-specialist can simply fabricate a membrane with a thickness of several micrometers using only PDMS and water. In addition, the membrane fabricated by FoW is significantly easier to handle, as water serves as the substrate and the fabricated membrane floats on the water surface. This reduces the preparation time wasted when the membrane is torn and refabricated. In addition, the FoW method is not limited to fabricating ‘flat’ membranes; alternative membrane structures are also possible by wisely using physical phenomena, such as surface tension or supportive structures. As examples, the fabrications of alternative membrane structures, such as the double-layered membrane, membrane-on-pins, and water capsule, were presented. Therefore, the FoW could be utilized in many applications, including microvalves, micropumps, microlenses, and cell culture substrates, which require microscale-thin membranes.

## Figures and Tables

**Table 1:** Four different experimental conditions, and an extra three experimental conditions with varying amounts of PDMS solution.

Experimental Conditions	Amount of PDMS Solution (μg)	Water Temperature (°C)	Initial PDMS Solution Temperature (°C)	Remarks
Condition 1	10	80	20	Control
Condition 2	20	80	20	Amount of PDMS solution is changed
Condition 3	10	40	20	Water temperature is changed
Condition 4	10	80	−15	Initial PDMS solution temperature is changed
Condition 5	30	80	20	Condition 5–7; extra experimental conditions with various amount of PDMS solution
Condition 6	40	80	20	
Condition 7	50	80	20	

## Supplementary Materials

The following are available online at https://www.mdpi.com/2073-4360/11/8/1264/s1, Figure S1: Images of support rings; Figure S2: Identifying membrane presence; Figure S3: PDMS wall surface tension; Figure S4: SEM images of multiple drops of PDMS; Figure S5: PDMS drops using various pipette tips; Figure S6: Insufficient PDMS amount; Figure S7: Membrane pull test; Videos S1 and S2: Speed camera videos of PDMS solution drop.
