# Supplementary material for: Floating-on-water Fabrication Method for Thin Polydimethylsiloxane Membranes
Source: Polymers (Basel). 2019 Jul 31;11(8):1264. doi: 10.3390/polym11081264 (PMC6722912; doi:10.3390/polym11081264)
Supplement: Supplementary file 1 [file polymers-11-01264-s001.pdf]

polymers

MDPI

Supplementary Materials: Floating-on-Water Fabrication Method for Thin Polydimethylsiloxane Membranes

Daehan Kim [1], Sung-Hwan Kim [1] and Joong Yull Park [1,*]

School of Mechanical Engineering, College of Engineering, Chung-Ang University, 84 Heukseok-ro, Dongjak-gu, Seoul 06974, Republic of Korea
* Correspondence: jrpark@cau.ac.kr; Tel.: +82-2-820-5888

Received: 8 July 2019; Accepted: 26 July 2019; Published: date

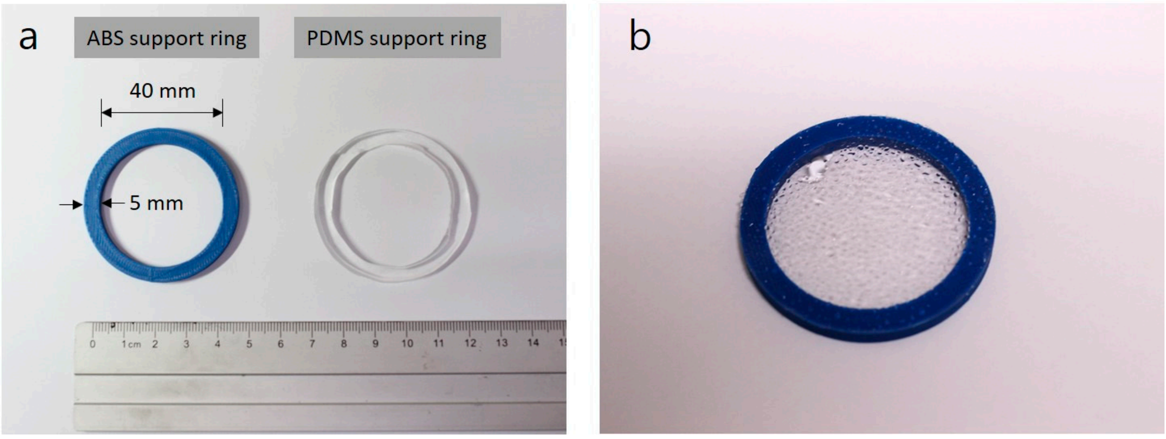

Figure S1. Supportive rings to handle fabricated membranes. (a) Support rings can be used to handle the membrane more conveniently. Example support rings shown here were fabricated of acrylonitrile butadiene styrene (ABS) or PDMS (; the inner diameter = 40 mm and the outer diameter = 50 mm). (b) The fabricated membrane was handled by using the ABS support ring. Water was sprayed over the membrane to ensure the presence of the membrane.

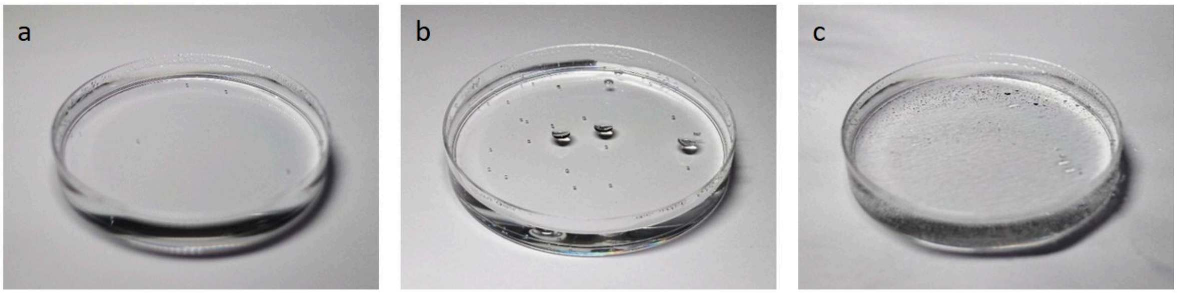

Figure S2. (a) It is difficult to distinguish whether the PDMS membrane is fabricated by the naked eye. Instead, it can be easily checked whether the membrane is formed or not by (b) dropping or (c) spraying water on the surface.

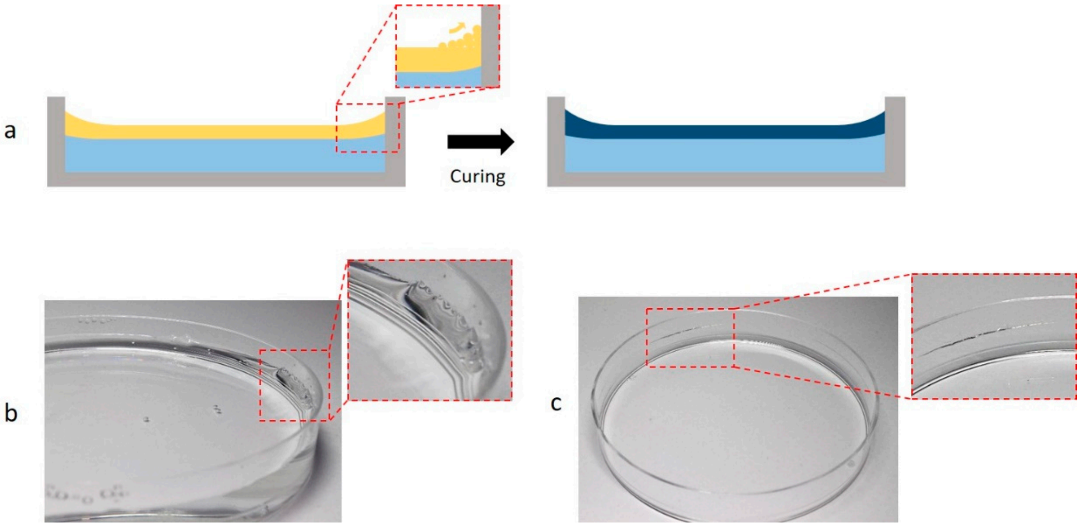

Figure S3. (a) The PDMS solution tends to climb up the wall by the surface tension at the point where the water surface and the wall meet. (b) The real image of the aggregated PDMS at the point where the water surface and the wall meet. (c) Residual PDMS leaves traces on the walls after removing the water and the membrane fabricated by FoW.

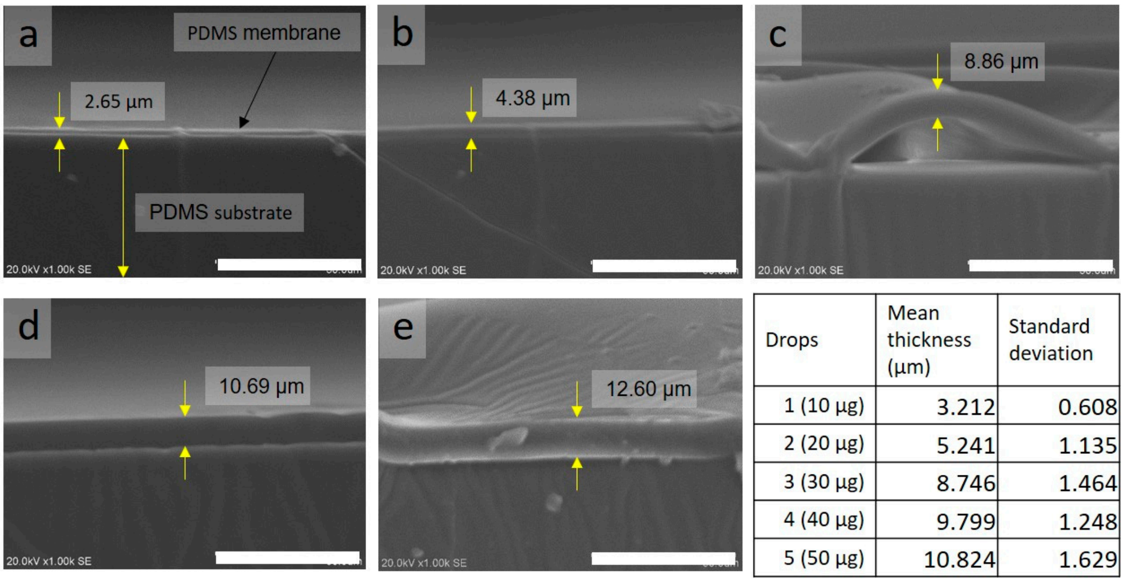

Drops	Mean thickness (µm)	Standard deviation
1 (10 µg)	3.212	0.608
2 (20 µg)	5.241	1.135
3 (30 µg)	8.746	1.464
4 (40 µg)	9.799	1.248
5 (50 µg)	10.824	1.629

Figure S4. SEM images of the PDMS membranes fabricated by using (a) 10, (b) 20, (c) 30, (d) 40, and (e) 50 µg of PDMS solution with the thickness values. The inset table shows the mean thickness and standard deviations calculated from $n = 5$ samples. The scale bar is 50 µm.

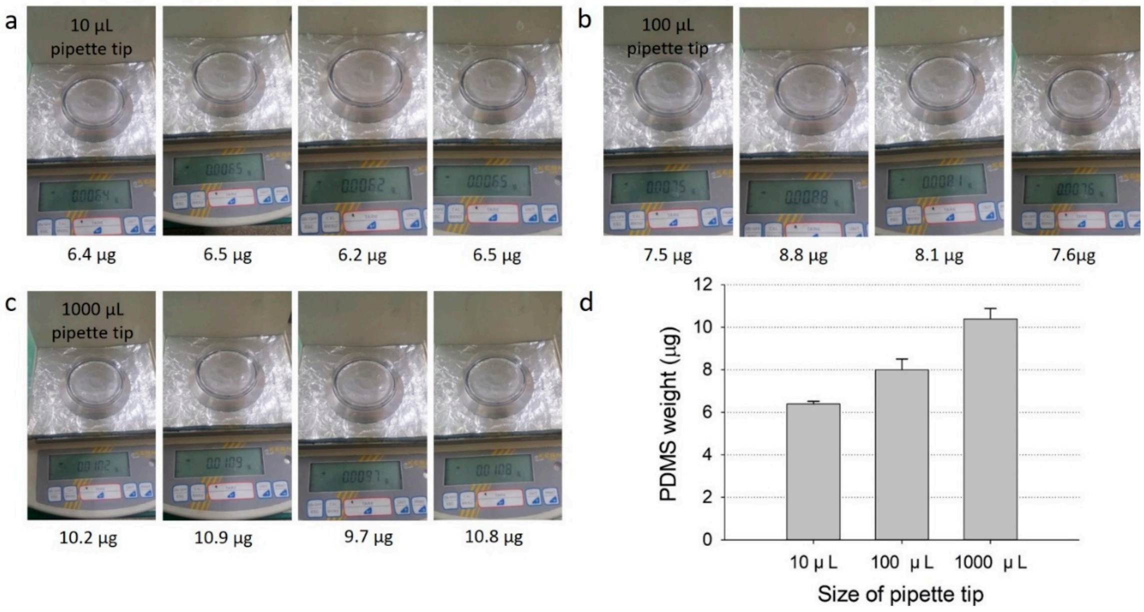

Figure S5. Repeated experiments were performed to accurately measure the weight of a drop of PDMS solution made from (a) 10, (b) 100, and (c) 1000 μL pipette tips. (d) The result data were gained from $n = 4$ samples.

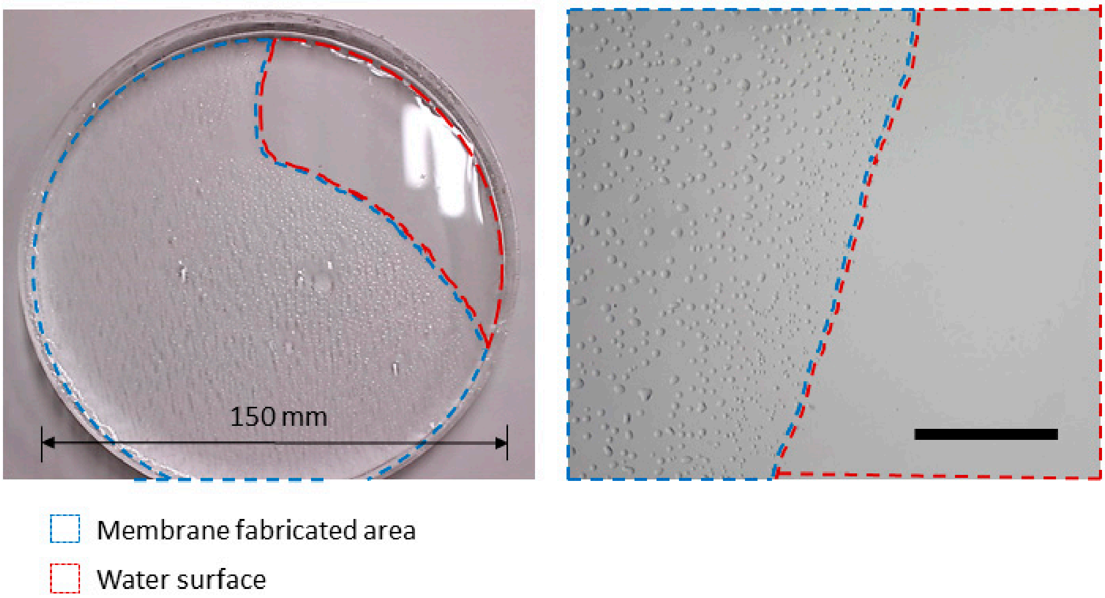

Figure S6. When the same amount of PDMS solution (10 μg) was used for 90 mm-diameter and 150 mm-diameter Petri dishes, a part of the water surface was not covered by the PDMS membrane in the 150 mm-diameter Petri dish. Membrane fabricated area was checked by spraying water on the surface. The scale bar is 4 mm.

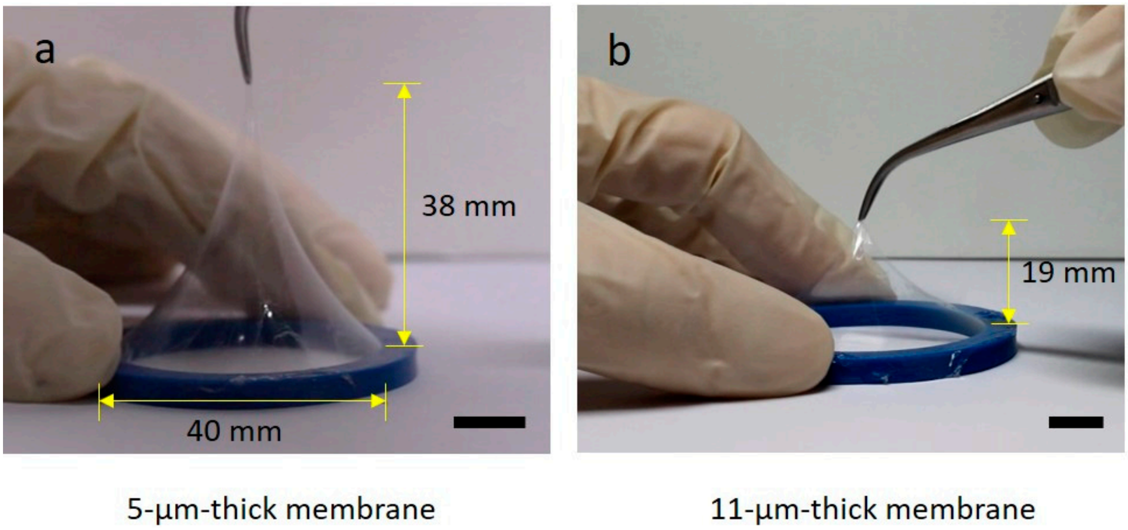

Figure S7. PDMS membrane specimens were pulled upward until breakage. The size of the support ring is the same as shown in Fig. S6. The scale bar is 10 mm. (a) The 5-µm-thick membrane was stretched by 38 mm before breakage and (b) the 11-µm-thick membrane was stretched only by 19 mm.

© 2019 by the authors. Submitted for possible open access publication under the terms and conditions of the Creative Commons Attribution (CC BY) license (http://creativecommons.org/licenses/by/4.0/).
